# Divergence in Male and Female Manipulative Behaviors with the Intensification of Metallurgy in Central Europe

**DOI:** 10.1371/journal.pone.0112116

**Published:** 2014-11-12

**Authors:** Alison A. Macintosh, Ron Pinhasi, Jay T. Stock

**Affiliations:** 1 PAVE Research Group, Department of Archaeology and Anthropology, University of Cambridge, Cambridge, United Kingdom; 2 Earth Institute and School of Archaeology, Newman Building, University College Dublin, Dublin, Ireland; Ohio State University, United States of America

## Abstract

Humeral morphology has been shown to reflect, in part, habitual manipulative behaviors in humans. Among Central European agricultural populations, long-term social change, increasing task specialization, and technological innovation all had the potential to impact patterns of habitual activity and upper limb asymmetry. However, systematic temporal change in the skeletal morphology of agricultural populations in this region has not been well-characterized. This study investigates diachronic patterns in humeral biomechanical properties and lengths among 174 adult Central European agriculturalists through the first ∼5400 years of farming in the region. Greater asymmetry in biomechanical properties was expected to accompany the introduction of metallurgy, particularly in males, while upper limb loading patterns were expected to be more similar between the Bronze and Iron Ages. Results revealed a divergence in the lateralization of upper limb biomechanical properties by sex between the Early/Middle Neolithic and Early/Middle Bronze Age. Neolithic females had significantly more variable properties than males in both humeri, while Bronze Age female properties became homogeneous and very symmetrical relative to the right-biased lateralization of contemporaneous males. The Bronze Age to Iron Age transition was associated with morphological change among females, with a significant increase in right-biased asymmetry and a concomitant reduction in sexual dimorphism. Relative to biomechanical properties, humeral length variation and asymmetry were low though some significant sexual dimorphism and temporal change was found. It was among females that the lateralization of humeral biomechanical properties, and variation within them, changed most profoundly through time. This suggests that the introduction of the ard and plow, metallurgical innovation, task specialization, and socioeconomic change through ∼5400 years of agriculture impacted upper limb loading in Central European women to a greater extent than men.

## Introduction

The biomechanics of the human upper limb are complex; largely free from weight bearing activities, the arms can be employed both unilaterally and bilaterally in a wide variety of ways. As the relationships between upper limb morphology, biomechanics, and manipulative behaviors are complex, interpreting morphology in the context of cultural change in the past can be challenging. A single dominant activity has the potential to obscure the influence of multiple less-dominant behaviors [1]–[13]. Yet, the habitual performance of a wide variety of behaviors, with no single dominant one, can also drive bone morphological change [14]. The interpretation of upper limb biomechanics following the transition to farming is particularly complex: technological developments, increasing socioeconomic complexity, and changing divisions of labor following the emergence of agriculture likely drove increasing diversity of manual activity at both the individual and population levels. In past agricultural populations, low humeral asymmetry in some individuals has been attributed to the bilateral loading associated with agricultural activities such as the grinding of grain and maize and/or the use of bimanual tools [15]–[19]. Yet marked lateralization has also been documented, which may be the result of unilateral loading associated with the manufacture and use of many stone, bone, and metal tools and weapons [17], [19]–[22]. Marked lateralization has also been documented in living agropastoralists performing physically demanding fieldwork from adolescence without mechanization [23].

Complexity in patterns of bone adaptation within and among agriculturalist groups is also associated with increasing social complexity through time. A comparison of two Italian agricultural populations with similar subsistence activities but different socioeconomic structure (Neolithic (∼6000-5500 BP) versus Iron Age (∼2600-2400 BP)) found marked humeral asymmetry in Iron Age males, associated with the use of weapons, but symmetry in Iron Age females, likely associated with the performance of cereal-processing activities [21]. In the Americas, pronounced sexual dimorphism in upper limb diaphyseal morphology has been noted during agricultural intensification, with male and female behaviors following different trends due to the sexual division of labor and/or social stratification [24]–[25].

The study of bone adaptation in modern human athletes of known loading regime has greatly facilitated the interpretation of complex behavior patterns in the past from skeletal remains. Many studies of athletes in racquet and throwing sports that produce bilaterally asymmetrical loading patterns have found greater bone mineral content, cortical area, total subperiosteal area, and bending and torsional rigidity in the dominant (loaded) limb relative to the non-dominant limb [1]–[13]. In contrast, bilateral upper limb loading in swimmers has been shown to increase diaphyseal cross-sectional robusticity relative to controls in both limbs and is associated with more equal bilateral humeral robusticity [12]. Bone length has a more limited responsiveness to loading than do diaphyseal cross-sectional dimensions [26]–[31], as it is more genetically canalized and has a limited period during which adaptation is possible (prior to growth plate closure) [30], [32]–[33]. Thus, evidence of adaptation in length asymmetry in response to upper limb loading is not as strong as that for diaphyseal adaptation. Krahl and colleagues [2] found significant lateralization in forearm length and bone diameters in professional tennis players that was not present in controls, but Haapasalo and colleagues [5] found no significant difference in length asymmetry between young tennis players and controls, so loading differences between young players and controls did not appear to be impacting bone length.

Expressing an individual's left and right bone biomechanical properties as percent asymmetries allows asymmetry to be more confidently attributed to the local influences of mechanical loading [34] and removes individual differences in systemic genetic and hormonal factors that may also affect bone morphology. This is particularly important when comparing the sexes, as pubertal testosterone and estrogen drive sex-specific change in the relative rates of periosteal and endosteal bone deposition and resorption [35]–[40], in muscle size/strength [10], [29], [41]–[42], and in the timing of growth plate closure [43]–[46], all factors which can affect bone length and/or cross-sectional dimensions.

There have been very few studies of asymmetry in humeral biomechanical properties or lengths following the introduction of farming, though see [47]. In Central Europe, variation in the complexity of grave assemblages and the degree to which cemeteries have been extensively studied and published means that more can be gleaned about possible behaviors during life in some of the region's cemeteries than in others. However, the existing Central European archaeological and osteological evidence suggests that changing technologies and divisions of labor had the potential to impact upper limb loading and asymmetry through time, particularly between the sexes.

### Central Europe from ∼5500 BC to ∼100AD

In Central Europe, the earliest farmers belonged to the Early Neolithic Linear Pottery cultures. In much of the region, this was the *Linearbandkeramik* (LBK) culture (∼5500-4900 BC), characterized by its distinctive pottery style, adze-axe stone tools, and timber longhouses [48]. LBK farmers practiced mixed farming, raising domesticated livestock and cultivating and harvesting cereals prior to metallurgy and mechanization, instead relying on manual tools like digging sticks and flint sickles [48]. In the Great Hungarian Plain, east of the Danube in Hungary, the earliest farmers belonged to the Alföld Linear Pottery (ALP) culture, or *alföldi vonaldiszes kerámia*, who shared many cultural features with the LBK [49].

Sex differences are evident in the dentition of LBK groups, indicative of dietary differences [49] and at least some division of tasks by sex [50]. For instance, older LBK females from the well-studied settlement and cemetery at Vedrovice (Moravia, Czech Republic) [51]–[66] as well as Nitra Horné Krškany (western Slovakia) [63], [66] show dental evidence of having used their teeth as tools, possibly for the working of plant fibers and the production of cord/rope [50], [67]. Pottery is also more often found with female remains in the Central European Neolithic [68]. In contrast, LBK males at Schwetzingen (Baden-Württemberg, Germany) [69] were more likely than females to be buried with flint arrowheads [70]. Stone adzes and axes, likely primarily used for woodworking, are found almost exclusively in male graves in Central Europe, including at Vedrovice, Schwetzingen, and Nitra Horné Krškany [63], [68]. Not only is the production of adzes/axes labor-intensive [63], their unilateral use produces similar upper limb movements and levels of strain as overhead throwing [71]. These sex differences in grave goods suggest that manipulative activities may have been quite different between Neolithic men and women in ways that may be reflected in upper limb asymmetry.

The many innovations in technology and metallurgy of the Middle/Late Neolithic likely also impacted upper limb loading. Simple ards were used for cutting shallow furrows in the soil, but bilateral manual labor with hoes and other implements would still have been required to first clear the land of brush or cut deeper furrows if required. Plows that actually turned the soil both allowed for more difficult soils to be worked and more land to be exploited [68], likely altering the manipulative behaviors associated with farming. Ard marks (long thin scratches) dating to the Middle Neolithic have been found in Central Europe [68], [72], while plow marks become prevalent by the end of the Middle Neolithic, ∼3500 BC [68], [73]–[74]. Early copper mining and metallurgy likely also had profound impacts on upper limb loading patterns among males and females. There was already evidence of copper metallurgy in Middle Neolithic Vinča settlement and burial contexts (∼5000-4460 cal BC) [75], and some Central European sites show evidence of the mining and smelting of copper ore [68], including Rudna Glava in Serbia [76]. These activities involved many unilaterally and bilaterally strenuous tasks with the upper limbs, such as the hammering and chiseling of ores out of the surrounding rock and their pounding and grinding prior to smelting [77].

Throughout the Bronze Age in Central Europe, in addition to the physical requirements of mining and the production of metal objects, the increased use of weaponry and the great variety of tasks in which individuals would have specialized all had the potential to produce differences in upper limb loading relative to the preceding Neolithic. By the Early Bronze Age (EBA; ∼2300-1500 cal BC) in Central Europe, metallurgy had intensified substantially and continued to do so through the Late Bronze Age (LBA: ∼1300-750 cal BC), with significant technological and socioeconomic consequences [78]–[79]. At the Early Bronze Age site of Brno-Tuřany (Moravia, Czech Republic), several individuals buried in reserve or storage pits on the settlement outskirts, rather than the more typical location in graves or settlement features, showed signs of impaired health or trauma, suggesting an influence of social stratification on burial practices [80]; however, subsequent analyses did not find any direct relationship between burial treatment and health status at the site [81].

Throughout the Bronze Age, copper metallurgy continued, with the novel addition of tin or arsenic to produce bronze, and the manual activities involved in copper and bronze production would have been similar. Agriculture also remained of primary importance in the Bronze Age, though upper limb loading patterns in the portion of the population involved in farming may have been altered by the use of ards and plows, querns for grinding grain, pounders, sickles, and various other implements at this time [82]. Additionally, the rise in prominence of warfare in the Bronze Age may have been associated with temporal and sex differences in upper limb loading. A wide range of weapons are found in the archaeological record at this time, including bows, arrowheads, daggers, halberds, swords, and spears, accompanied by the development of defensive armor and the presence of defended settlements [83]. Weapons are much more common in male graves than female graves in the Bronze Age, for example [84], so their production and/or use were probably more often male rather than female activities. The Bronze Age also brought more pronounced social stratification and complexity [73], [83], [85]–[86], social changes that are reflected in both the number and prestige of grave goods in some Central European cemeteries, for example [84], [87]. Bronze Age craft specialization was significant, involving not just the production of metal objects but of pottery, glass, and salt, as well as carpentry, the working of leather, bone, and antler, and the manufacture of textiles from wool and flax [79], [83], [88]. However, the manipulative activities involved in many of these crafts would have been similar, as would those involved in the quarrying and preparation of ores, stone, and flint [88].

The shifting role of metals in society documents a gradual transition between bronze and iron production. In the Early Iron Age (∼800-450 BC) [89], bronze was still used for weapons and tools, but as the use of iron in tool manufacture increased, bronze remained important for personal ornamentation and various utilitarian items. Though the types of items made from bronze shifted in the Iron Age, the manipulative activities involved in its mining, smelting, and smithing would have remained similar [89]. The mining of iron would not have required much change in method or upper limb loading; however, once iron tools could be used, overall efficiency likely increased significantly [77]. Iron was stronger and more widely available than bronze, so could be used to produce an exceptional variety of tools. Its use in agricultural implements such as plows, scythes, shovels, and hoes also greatly increased the efficiency of food production and harvesting [89]. In addition, the ability to work iron freed communities from a dependence on more distant and less readily available sources of other metals typical of the Bronze Age, as iron ore deposits were ubiquitous and sizeable [77]. Large fortified settlement centers of significant political and commercial importance formed in the Iron Age [77], and craft specialization and socioeconomic stratification were high [90]. Crafts were diverse, including the working of a variety of metals and the production of pottery [89], and their assignment would likely have been sex-specific. Warfare was an important part of Iron Age society in Central Europe, particularly for men, with a variety of weapons being produced and found in large numbers in burial, ritual, and settlement contexts [89]. High social stratification may be interpreted from differential funerary treatment, access to dietary resources, and stature among Iron Age Celtic and Scythian males [91]–[92] and health differences among Scythian men and women (northeast Hungary) [93]. However, in terms of upper limb loading, the wide variety of tasks in which Iron Age individuals were involved, combined with high social complexity, likely means that variation in the degree to which any one pattern of strenuous upper limb bone loading predominated may be reduced relative to earlier groups.

### Central European humeral biomechanics

Given the evidence for considerable social and technological change following the introduction of agriculture in Central Europe, it is likely that the type or distribution of habitual manipulative behaviors among members of the population would have been affected. Behavioral change may have altered upper limb loading, and thus may be reflected in humeral morphology. The mechanical performance of limb bones can be quantified by the calculation of cross-sectional geometric (CSG) properties, including estimates of compressive strength (total subperiosteal area; *TA*) and bending and torsional rigidity (polar second moment of area; *J*) [34], [94]. To date there has been only one published study of temporal change in humeral biomechanics through this time in past Central European populations. Sládek and colleagues [47] compared humeral length, cross-sectional strength, and shape in Late Eneolithic (copper metallurgy) and EBA groups in Lower Austria, Moravia, and Bohemia. The authors found that the manipulative behaviors associated with both copper and bronze metallurgy in this region were similar, with no significant change in humeral robusticity or its patterns of asymmetry in either sex. In both time periods, humeral cross-sectional morphology indicated asymmetrical right-biased loading in males but little asymmetry in maximum length, while the situation was reversed in females, with very symmetrical loading between left and right humeri but right-biased length lateralization. This reversed lateralization in length and diaphyseal CSG properties between the sexes in Central Europe is consistent with Auerbach and Ruff's [30] findings in a larger sample of Holocene adults, which included 151 Neolithic, Bronze Age, Iron Age, and Early Medieval Europeans.

The current study attempts to elucidate the long-term effects of agricultural intensification, the introduction and expansion of metallurgy, and social change on habitual upper limb behaviors during the first ∼5400 years of agriculture in Central Europe. To do so, asymmetry and variability in humeral maximum lengths and CSG properties are compared temporally and between the sexes. Asymmetry and variability are expected to be higher in CSG properties than in maximum lengths in all time periods. It is expected that significant differences in upper limb asymmetry and variability will be found between the Early/Middle Neolithic and Early/Middle Bronze Age groups, associated with greater agricultural efficiency, the expansion of mining and copper and bronze metallurgy, the manufacture and production of metal objects and other crafts, and the increased task specialization that accompanied these changes. Given the considerable overlap of bronze and iron production in Central Europe [90], [95], reduced temporal change in humeral asymmetry between the Early/Middle Bronze Age and Iron Age groups is expected.

## Materials and Methods

### Skeletal sample

**Figure 1:**
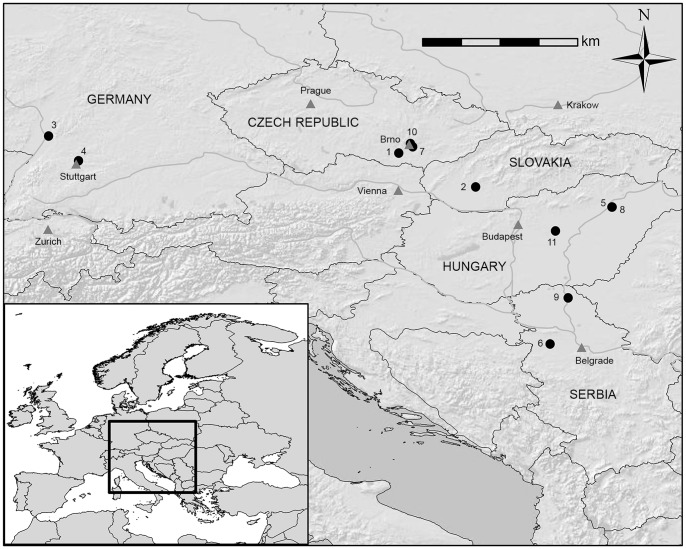
Map of Central Europe with geographical location of cemeteries: 1. Vedrovice 2. Nitra Horné Krškany 3. Schwetzingen 4. Stuttgart-Mühlhausen 5. Polgár-Ferenci-hát 6. Hrtkovci-Gomolava 7. Brno-Tuřany 8. Polgár Kenderföld 9. Ostojićevo 10. Brno-Maloměřice 11. Tápiószele.

All skeletal remains utilized for this research are from archaeological populations with broadly similar subsistence patterns, with primary reliance on domesticated crops and livestock [48], representing portions of three archaeological time periods following the transition to agriculture: the Early/Middle Neolithic (∼5300-4600 cal BC), Early/Middle Bronze Age (∼2300-1450 BC), and Early through Late Iron Age (∼850BC-100 AD). All remains were originally excavated from southwest Germany, western Slovakia, Hungary, the Czech Republic, and northern Serbia (Fig. 1) and are housed in museum and university collections. No permits were required for the described study, which complied with all relevant regulations. Sample details on all Central European cemeteries included in analyses are available in Table 1, and more detailed specimen information and all relevant data are presented in Tables S1 and S2.

**Table 1:** Central European sample details.

Time Period and Culture	ApproximateDate (BC)*	Cemetery	Cemetery Location	Collection Housed At:	Individuals (males/females)
**Neolithic**					**83 (49/34)**
*Early*					
LBK	5300–5100*	Vedrovice	Czech Republic	Moravian Museum (Brno)	18 (6/12)
LBK	5370–4980*	Nitra Horné Krškany	Slovakia	Moravian Museum (Brno)	12 (7/5)
LBK	5260–5010*	Schwetzingen	Germany	Stuttgart Regional Council, State Conservation Office- Osteology (Konstanz)	13 (8/5)
LBK	5200–4960*	Stuttgart-Mühlhausen	Germany	University of Tübingen	22 (13/9)
ALP	5300–5070*	Polgár-Ferenci-hát	Hungary	Hungarian Natural History Museum (Budapest)	8 (6/2)
*Middle*					
Vinča	∼4950–4600*	Hrtkovci-Gomolava	Serbia	Museum of Vojvodina (Novi Sad)	10 (9/1)
**Bronze Age**					**62 (34/28)**
*Early*					
Únětice	2300–1700	Brno-Tuřany	Czech Republic	Masaryk University (Brno)	12 (8/4)
Maros	∼1600/1500	Ostojićevo	Serbia	National Museum of Kikinda	33 (15/18)
*Middle*					
Füzesabony	1550–1450	Polgár Kenderföld	Hungary	Hungarian Natural History Museum (Budapest)	17 (11/6)
**Iron Age**					**35 (14/21)**
*Early*					
Bosut	850–600/500	Hrtkovci-Gomolava	Serbia	Museum of Vojvodina (Novi Sad)	6 (3/3)
*Middle*					
Celtic	400–200	Brno-Maloměřice	Czech Republic	Moravian Museum (Brno)	14 (4/10)
*Late*					
Scythian	385-100AD*	Tápiószele	Hungary	Hungarian Natural History Museum (Budapest)	15 (7/8)

* indicates calibrated radiocarbon date; *N* =  number of individuals; LBK =  Linearbandkeramik; ALP =  Alföld Linear Pottery; dates from: [49, 51 57, 62, 93, 95, 117–118, 134, 147], Zdeněk Tvrdý, pers. comm.

A total of 174 individuals (96 males, 78 females) had sufficient preservation of at least one humerus to be included in analyses of variation in length and CSG properties. Of these, 156 (85 males, 71 females) had paired elements from which asymmetry in CSG properties could be calculated. In 22 individuals, poor preservation of proximal or distal joint surfaces in one of the paired humeri required the estimation of maximum length from the well-preserved side, leaving 134 pairs of humeri (73 males, 61 females) for inclusion in analyses of humeral length asymmetry.

Age was estimated and sexes determined according to the methods outlined in Buikstra and Ubelaker [96]. In order to reduce the effects of age-related differences in cortical thickness between individuals on mechanical property estimates [28], only skeletally mature adults were included in analyses, with preference given to adults within the approximate age range of ∼20–40 years. However, limitations of sample size and preservation status meant that this was not always possible.

### Silicone moulding and quantification of CSG properties

CSG properties of left and right humeri were generated using a silicone moulding method [97]–[98]. Periosteal moulds were taken at 35% of maximum humeral length using Coltène President polyvinyl siloxane putty. Estimates of CSG properties from periosteal methods have shown a strong correspondence with true CSG properties derived from both the periosteal and endosteal contour at the 35% location [98]. In addition, this location has been used to show strong differences in upper limb loading between human groups [17]–[18], [99], though it should be noted that the full range of elbow loading in humans might not necessarily be reflected at this location [20]. Moulds were then scanned in anatomical orientation using a flatbed document scanner, orientated with the x-axis running mediolaterally and the y-axis running anteroposteriorly. Mould images were imported into Adobe Photoshop and periosteal contours were traced, creating a solid cross-sectional image of the humeral shaft. CSG properties were quantified using BoneJ [100], a bone image analysis plug-in for ImageJ (http://rsbweb.nih.gov/ij/). Periosteal mould images do not provide visualization of the endosteal contour; however, periosteally-derived CSG properties (such as those quantified from silicone moulding and laser surface scanning) have shown strong correspondence with true CSG properties [98], [101]–[103].

The CSG properties analyzed in this study are total subperiosteal area (*TA*) and the polar second moment of area (*J*). *TA* is highly correlated with cortical area and provides a measure of compressive strength (mm^2^) [34]. *J* provides an estimate of torsional and (twice) average bending rigidity in any two perpendicular planes through the sum of the second moments of area for those planes, for instance about anteroposterior (*I_x_*) and mediolateral (*I_y_*) or maximum (*I_max_*) and minimum (*I_min_*) planes [34]. In this instance, *J* was quantified as the sum of *I_max_* and *I_min_*. Size-standardization of CSG properties was performed following the method of Ruff [34]: *TA*/estimated body mass and *J*/(estimated body mass * maximum bone length^2^). Maximum humeral length parallel to the long axis of the diaphysis was recorded using an osteometric board, and body mass was estimated using an average of left and right femoral head diameters (obtained using sliding calipers) following the equations for European Holocene populations presented by Ruff and colleagues [104].

### Quantification of humeral asymmetry and variability

Humeral asymmetries in maximum length and CSG properties were explored through the conversion of lengths and unstandardized *TA* and *J* values into percent directional asymmetries (%DA): ((right-left)/(average of left and right))*100 [30], [105]–[106]. Percent directional asymmetry provides a measure of both the magnitude of asymmetry and its direction: positive %DA indicates relative hypertrophy in the right humerus compared to the left, and vice versa when %DA is negative. Following Stock and colleagues [107], a 0% cut-off was used to determine right-bias frequencies for *TA* and *J*; Auerbach and Ruff [30] showed that similar results were found when 0%, 0.5%, or 1% DA was used as a cut-off distinguishing handedness from fluctuating asymmetry. However, three individuals with very low asymmetry (one Iron Age Brno-Maloměřice male, and two Bronze Age females from Ostojićevo and Brno-Tuřany) were slightly negative in one property but slightly positive in the other, resulting in different percentages of right-bias for *TA* and *J*. Variability in maximum length, *TA*, and *J* in both left and right humeri by sex was quantified through the calculation of coefficients of variation (CV), calculated as (standard deviation/mean) *100, which provide a size-independent method for the evaluation of relative variation [108].

### Statistical analyses

The Kruskal-Wallis test was used examine differences in %DA in maximum length, *TA*, and *J* in each sex between time periods as well as between cemeteries within each time period. Mann-Whitney tests were also used to test for sex differences in asymmetry in humeral length, *TA*, and *J* within each time period. A modified Levene's test for homogeneity of mean-adjusted absolute deviation scores was used to test for differences in CVs through time and between the sexes, as these might be indicative of changing task specialization and the range of activities being performed among males and females. The total group mean for each variable was calculated for males and females in each time period. Each individual's value for each variable, for example *TA*, was then adjusted by his or her group mean using the following equation, where ABS refers to absolute deviation: ((ABS(*TA* - total group mean for *TA*))/total group mean for *TA*). The result of this equation was an absolute deviation value for each individual, indicating how far each individual's value was from the group mean for a given variable (in this example, *TA*). One-way analysis of variance (ANOVA) was used to test for temporal change in these absolute deviation scores adjusted for the mean, in each sex as well as between the sexes. If the ANOVA indicated a significant difference between the absolute deviation scores of two groups, variation between the groups was considered to be significantly different.

To determine whether or not the percentage of right-biased individuals for *TA* and *J* in each time period was significantly different from what would be expected by chance, chi-squared tests were used. Due to the need for both humeral elements to be well preserved, sample sizes were reduced for the examination of asymmetry. For this reason, no further statistical analyses were run on percent right-bias data. All statistical analyses were conducted in SPSS v20.

## Results

### Lateralization in humeral length, *TA*, and *J*

**Figure 2:**
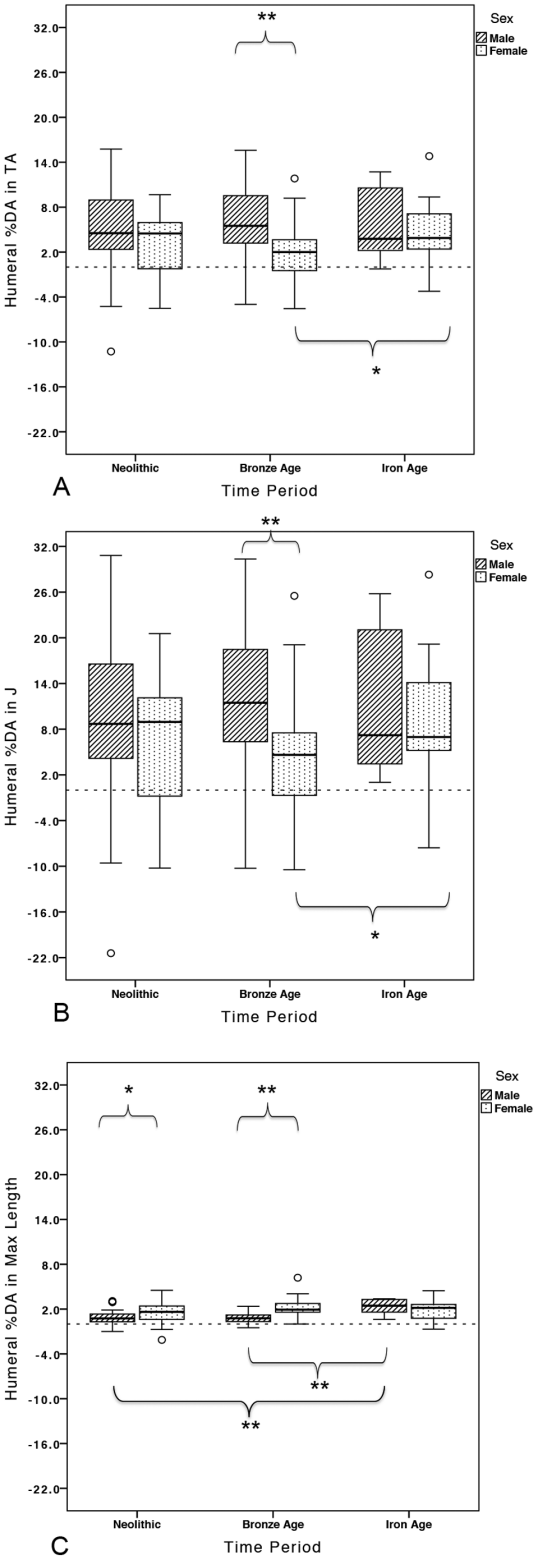
Percent directional asymmetry in humeral (A) *TA*, (B) *J*, and (C) maximum length by time period and sex. Brackets indicate significant differences (*p*<0.05 denoted by *; *p*<0.001 denoted by **).

**Figure 3:**
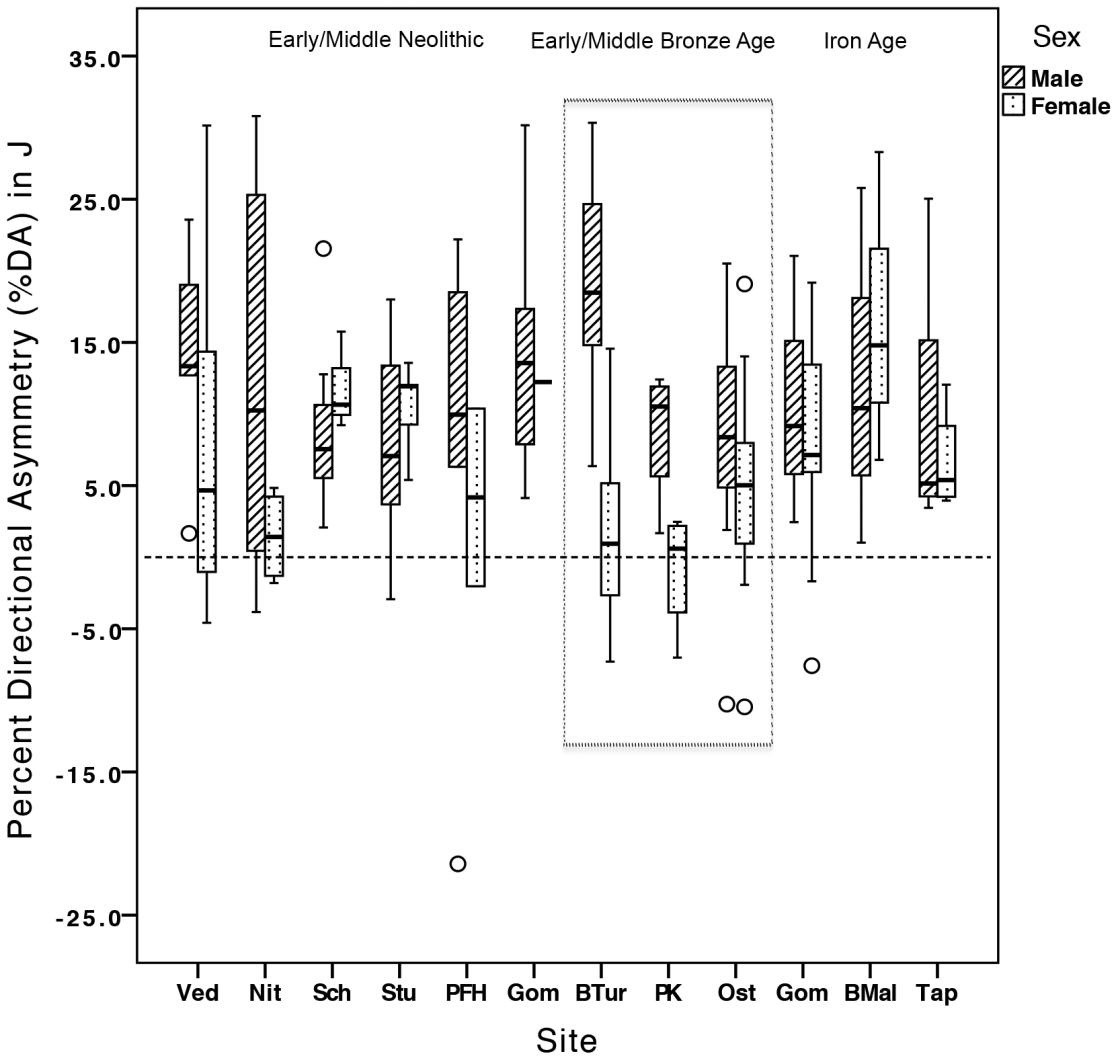
Percent directional asymmetry in humeral *J* by cemetery. Sites  =  Ved: Vedrovice; Nit: Nitra Horné Krškany; Sch: Schwetzingen; Stu: Stuttgart-Mühlhausen; PFH: Polgár-Ferenci-hát; Gom: Hrtkovci-Gomolava (Vinča); BTur: Brno-Tuřany; PK: Polgár Kenderföld; Ost: Ostojićevo; Gom: Hrtkovci-Gomolava (Bosut); BMal: Brno-Maloměřice; Tap: Tápiószele.

Summary statistics for mean %DAs in humeral length, *TA*, and *J* by time period and sex are presented in Table 2, and by cemetery and sex in Table 3. Among females, %DA for both *TA* and *J* increased significantly from the Bronze Age to the Iron Age (*TA*: *p*<0.034, *J*: *p<*0.038; Fig. 2a,b). Low levels of asymmetry among Bronze Age females are in marked opposition to high lateralization among Bronze Age males, driving pronounced sexual dimorphism in %DA in the Bronze Age group overall, in both *TA* and *J* (*p<*0.001 for both). Among Bronze Age cemeteries, it was the EBA cemetery of Brno-Tuřany that particularly drove high lateralization in *J* among males: at this cemetery, males had significantly higher %DA in *J* (%DA = 19.16%) than males at either Polgár Kenderföld (%DA = 8.78%; *p*<0.038) or Ostojićevo (%DA = 8.79%; *p*<0.024) (Fig. 3). Percent DA in maximum length also increased significantly in the Iron Age, in males relative to both Neolithic (*p<*0.006) and Bronze Age (*p*<0.004) males (Fig. 2c). This length asymmetry among Iron Age males led to a reduction in the high levels of sexual dimorphism in asymmetry noted among earlier groups: Neolithic and Bronze Age females had significantly higher %DA in maximum length than males (*p<*0.011 and *p*<0.001, respectively).

**Table 2:** Summary statistics for mean percent directional asymmetries (%DA) by time period and sex.

Time Period	Sex		*TA*		*J*			Max Length	
		N	Mean	SD	Mean	SD	N	Mean	SD
**Neolithic**	Males	50	5.24	5.09	10.21	9.98	41	0.78	0.98
	Females	29	3.65	4.32	7.29	8.50	28	**1.51**	1.44
**Bronze**	Males	25	**6.20**	4.68	**12.37**	9.28	26	0.74	0.70
	Females	26	2.04	4.01	4.25	8.04	21	**2.09**	1.42
**Iron Age**	Males	10	5.49	4.78	10.86	9.54	6	2.296 ^c^	1.098
	Females	16	4.61**^a^**	4.22	9.07**^b^**	8.38	12	1.855	1.423

Max Length =  maximum bone length in cms; underlined superscripts a and b indicate significantly higher %DA in Iron Age females relative to the Bronze Age (a: *p*<0.034; b: *p*<0.038); underlined superscript c indicates significantly higher %DA in Iron Age males relative to Neolithic (*p*<0.006) and Bronze Age (*p*<0.004) males; bold indicates significant sexual dimorphism (*TA* and *J*: p<0.001 for both; max length: Neolithic *p*<0.011; Bronze Age *p*<0.001).

**Table 3:** Summary statistics for mean percent directional asymmetries (%DA) by time period and sex.

Time Period	Site	Sex	Percent directional asymmetry (%DA)							
				*TA*		*J*			Max Length	
			N	Mean	SD	Mean	SD	N	Mean	SD
NEOLITHIC	Vedrovice	M	5	6.78	3.92	14.06	8.24	3	1.08	0.27
		F	11	3.86	5.55	7.83	11.0	7	1.23	1.74
	Nitra Horné Krškany	M	8	6.40	6.91	12.37	13.40	6	0.74	1.27
		F	4	1.33	2.56	1.46	3.26	4	1.26	0.73
	Schwetzingen	M	7	3.84	1.94	7.07	3.40	3	−0.38	0.67
		F	3	5.94	1.66	11.87	3.43	4	1.15	1.58
	Stuttgart-Mühlhausen	M	12	3.98	3.52	7.55	6.80	12	0.93	0.90
		F	7	5.13	1.33	10.50	2.77	10	1.73	1.21
	Polgár-Ferenci-hát	M	6	3.77	7.96	7.58	15.49	6	0.36	0.82
		F	2	2.04	5.53	4.17	8.78	2	2.08	3.44
	Hrtkovci-Gomolava	M	10	7.41	3.95	14.38	7.99	11	1.10	1.01
		F	1	5.94	-	12.23	-	1	2.49	-
BRONZE AGE	Brno-Tuřany	M	7	9.61	4.41	**19.16**	8.54	5	1.09	0.70
		F	5	0.99	4.31	2.14	8.32	3	1.90	1.61
	Polgár Kenderfold	M	4	4.49	2.47	**8.78**	4.88	10	0.65	0.86
		F	4	−0.56	2.19	−0.84	4.34	4	2.68	1.04
	Ostojićevo	M	13	4.36	4.14	**8.79**	8.36	11	0.65	0.53
		F	16	2.41	3.49	4.85	6.83	14	1.97	1.52
IRON AGE	Hrtkovci-Gomolava	M	3	5.58	4.62	10.88	9.41	3	2.76	0.73
		F	9	3.83	4.10	7.61	8.39	7	1.72	1.29
	Brno-Maloměřice	M	3	5.96	6.50	12.40	12.51	1	3.29	-
		F	3	8.33	5.99	16.63	10.9	0	-	-
	Tápiószele	M	4	5.08	5.07	9.70	10.26	2	1.10	0.68
		F	4	3.56	1.80	6.69	3.71	5	2.04	1.73

M =  male; F =  female; N =  number of paired humeri; Max Length =  maximum bone length in cms; bold indicates significant difference in %DA in *J* between Bronze Age males: Brno-Tuřany significantly higher than Ostojićevo (*p*<0.024) and Polgár Kenderfold (*p*<0.038).

**Table 2.** Summary statistics for mean percent directional asymmetries (%DA) by time period and sex.

**Table 3.** Summary statistics for percent directional asymmetries (%DAs) by cemetery and sex.

**Figure 4:**
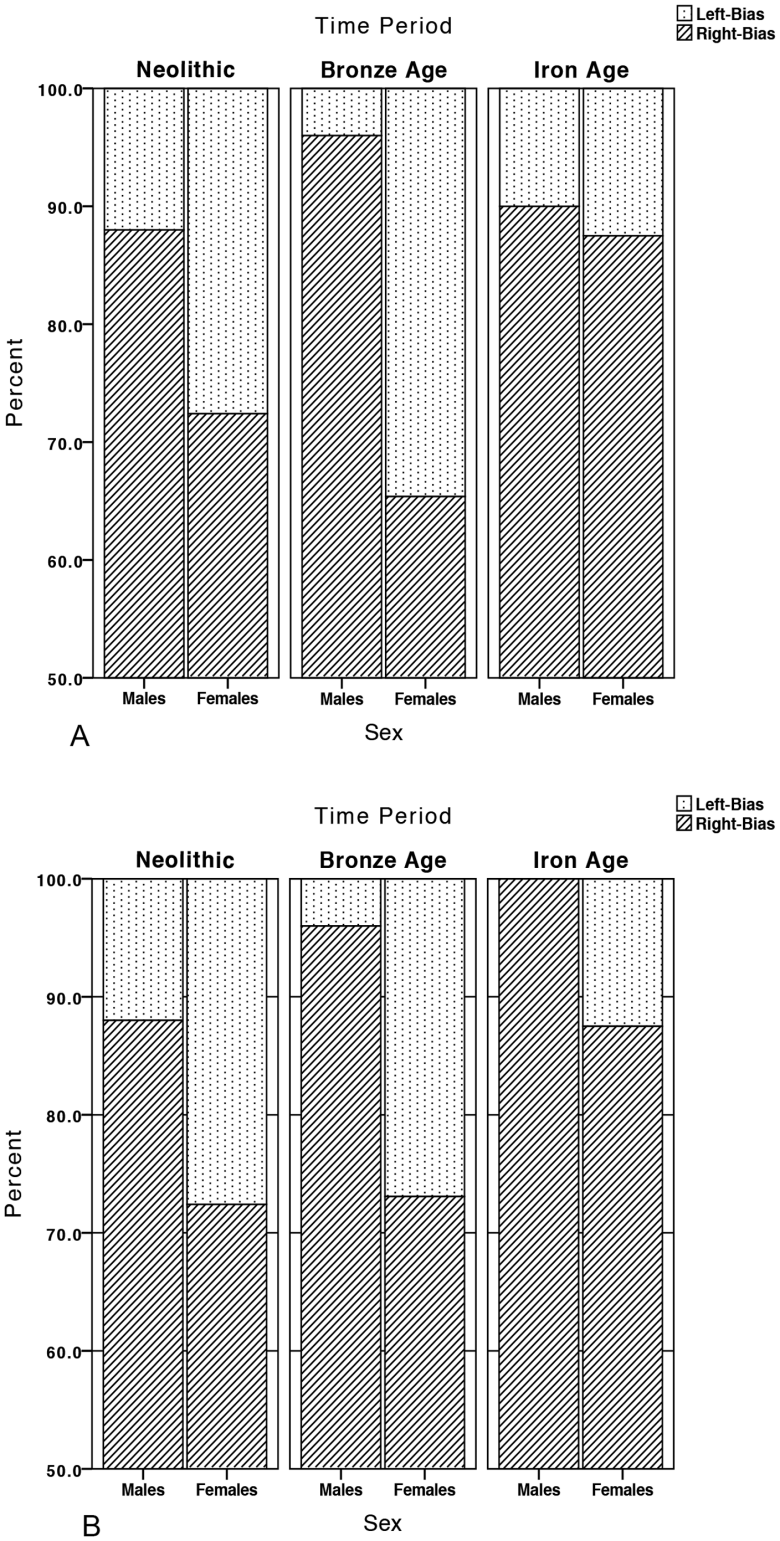
Percent left and right bias in (A) *TA* and (B) *J* by time period and sex.

Due to very low overall percent asymmetries in maximum length, only percent right bias in *TA* and *J* were calculated and together these provided a consistent picture through time of pervasive right-dominance in humeral strength and rigidity (Fig. 4a,b). Right and left bias frequencies in humeral *TA* and *J* by time period and sex are presented in Table 4. Right-biased humeral *TA* and *J* were significantly greater than would be expected by chance in both sexes in the Neolithic and Bronze Age and in Iron Age females, supporting a behavioral component to this lateralization.

**Table 4:** Right and left bias frequencies in humeral *TA* and *J* by time period and sex.

Time Period	Males					Females				
	N	R	L	%R	*p*	N	R	L	%R	*p*
***TA***										
Neolithic	50	44	6	88.00	<0.001	29	21	8	72.40	*<*0.016
Bronze	25	24	1	96.00	<0.001	26	17	9	65.40	*<*0.019
Iron Age	10	9	1	90.00	ns	16	14	2	87.50	<0.003
										
***J***										
Neolithic	50	44	6	88.00	<0.001	29	21	8	72.40	*<*0.016
Bronze	25	24	1	96.00	<0.001	26	19	7	73.10	*<*0.019
Iron Age	10	10	0	100.00	ns	16	14	2	87.50	<0.003

N =  total number of paired humeri; R =  number of individuals with right-dominance (+%DA);

L =  number of individuals with left-dominance (-%DA); *p* values reflect *X* ^2^ tests for significant;

right-biasing relative to what would be expected by chance; ns =  not significant at *p*<0.05.

### Variability in humeral length, *TA*, and *J*

**Figure 5:**
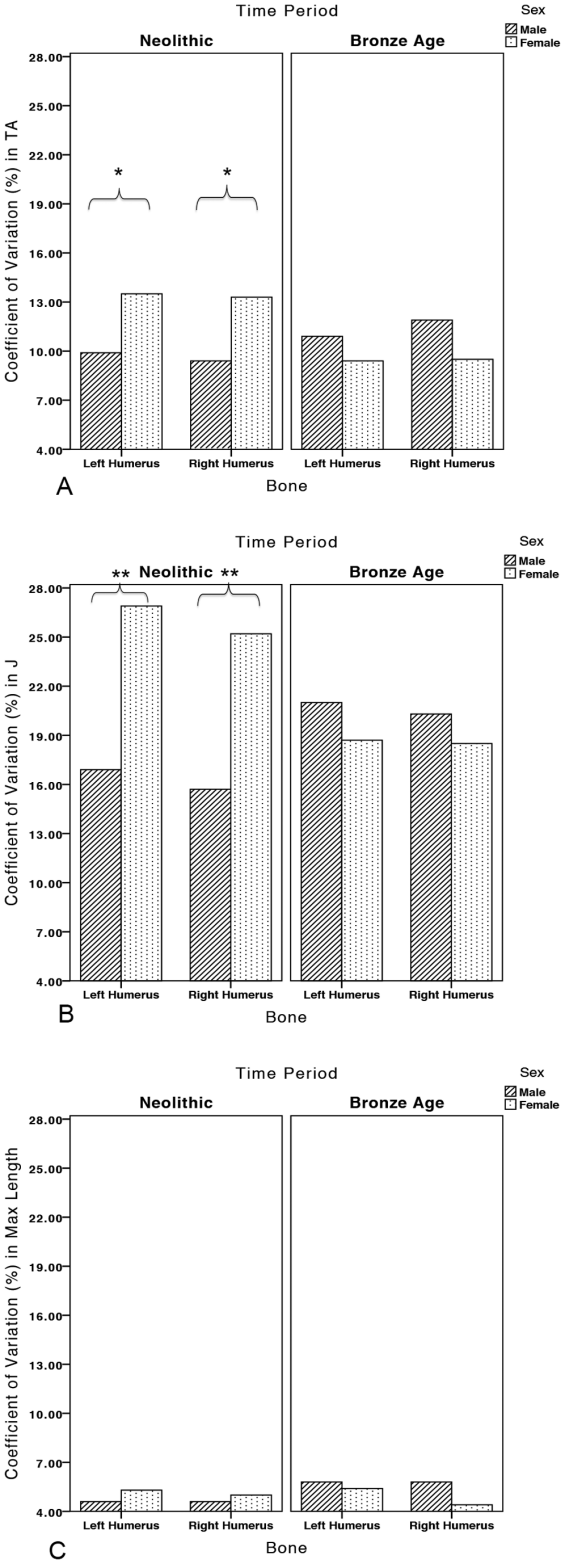
Coefficients of variation (%) in left and right humeral (A) *TA*, (B) *J*, and (C) maximum length in the Neolithic and Bronze Age periods by sex. Brackets indicate significant differences (*p*<0.05 denoted by *; *p*<0.001 denoted by **).

**Figure 6:**
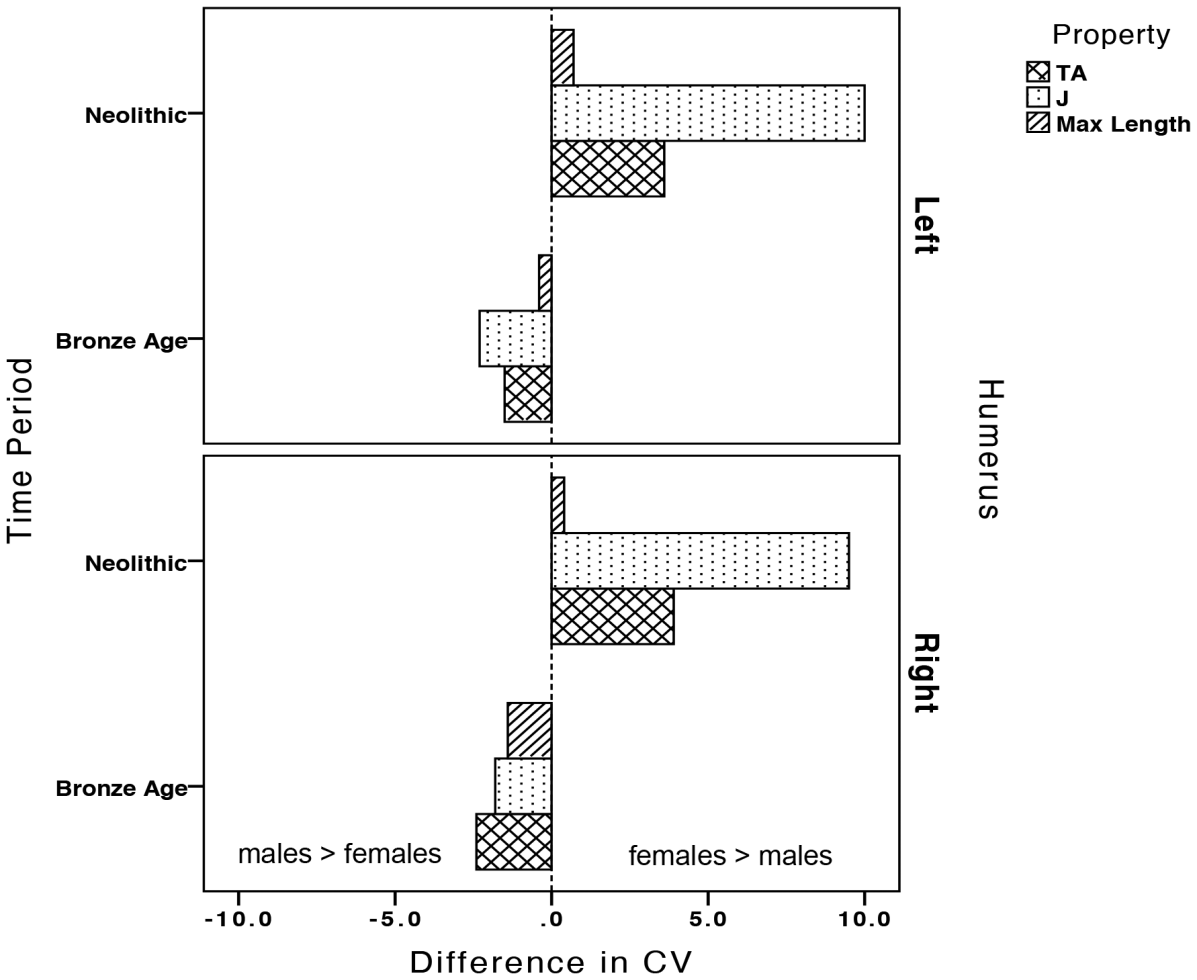
Change in the sexual dimorphism of coefficients of variation (CV) in left and right humeral properties between the Neolithic and Bronze Age periods, calculated as the difference between female and male CVs (female CV - male CV).

Coefficients of variation (CVs) in left and right humeral CSG properties and maximum length by time period and sex are presented in Table 5. No temporal change in the variability in any property was found in left or right humeri in either sex. However, compared to Neolithic males, Neolithic females had significantly more variable *TA* and *J* in both left and right humeri (Fig. 5a,b; *TA*: left humerus p<0.047, right humerus p<0.015; *J*: left humerus p<0.006, right humerus p<0.001). Neolithic females also had slightly higher CVs for maximum length than males, but not significantly so (*p = *0.748 for left humeri, *p* = 0.842 for right humeri; Fig. 5c). Though there was no significant sexual dimorphism in variability in the Bronze Age, the pattern of variability between the sexes reversed relative to the Neolithic (Fig. 5); Bronze Age males became more variable than females in all properties in both humeri (Fig. 6).

**Table 5:** Humeral coefficients of variation (CVs) by time period and sex.

Time Period	Coefficient of Variation (%)										
	Left Humerus						Right Humerus				
	N	*TA*	*J*	N	ML	N		*TA*	*J*	N	ML
**Neolithic**											
Males	48	9.90	16.90	63	4.60	48		9.40	15.70	61	4.60
Females	32	**13.50**	**26.90**	40	5.30	30		**13.30**	**25.20**	37	5.00
**Bronze Age**											
Males	33	10.90	21.00	42	5.80	28		11.90	20.30	42	5.80
Females	27	9.40	18.70	33	5.40	28		9.50	18.50	32	4.40
**Iron Age**											
Males	12	13.70	22.30	26	5.00	11		10.50	18.20	28	4.80
Females	20	12.20	21.80	23	4.90	16		11.70	19.30	20	4.80

N =  total number of humeri; ML =  maximum bone length in cms; bold indicates significant sexual dimorphism in CV.

(Neolithic *TA*: left humerus *p*<0.047, right humerus *p*<0.015; Neolithic *J*: left humerus *p*<0.006, right humerus *p*<0.001).

## Discussion

### Neolithic through Bronze Age transition

This study explores the impact of agricultural intensification, metallurgy, and social change on habitual manipulative behaviors through ∼5400 years of farming in Central Europe. Mean asymmetry in *TA* or *J* was not significantly different between the sexes in the Early/Middle Neolithic. The range of %DAs among Neolithic males and females exhibited considerable overlap, but Neolithic females had significantly more variable CSG properties in both left and right humeri than males. Thus, results suggest that Neolithic females were loading their upper limbs in a wider variety of ways than males, likely through a broader range of manual activities, and that these activities generated similar patterns of lateralization to males (no significant differences in %DA). Women at the earliest LBK cemetery in Moravia (Czech Republic), Vedrovice, displayed a wide range of humeral %DAs, while mean female %DA values at the two early LBK cemeteries from southwest Germany, Schwetzingen [69], [109]–[110] and Stuttgart-Mühlhausen [111], were particularly high, especially relative to males at these sites (see Fig. 3). Early Neolithic females likely participated in a range of bilateral and unilateral manipulative activities, related to crop planting/harvesting and the grinding of grain, gardening, the tending of livestock, the production of pottery and personal ornamentation, and the working of plant fibers for rope/cord (based on manipulative tooth wear) [50]–[51]. Many Neolithic males and females exhibited left-biased or symmetrical %DA values (see Fig. 2a,b), which could be related to the two-handed use of tools for grinding grain and/or digging implements and hoes that would have loaded both limbs. The use of bimanual digging tools and hoes may even have required greater loading of the non-dominant limb, similar to the distribution of upper limb loading in bimanual spear use [112]–[113].

A small proportion of Neolithic males in the current study had very high %DA levels outside the range of Neolithic females (Fig. 2a,b). Thus, at least some males were performing tasks that heavily loaded their right humerus relative to their left, to an extent not seen in females. Given that the stone adze-axe is found almost exclusively in male graves in the Neolithic of Central Europe [63], [66], construction and woodworking utilizing these tools were likely unilateral tasks more typically performed by Early Neolithic males. Particularly high %DAs among Neolithic males were found in two early LBK settlements, Vedrovice in Moravia (Czech Republic) and Nitra Horné Krškany in western Slovakia, and the only Middle Neolithic cemetery included, Gomolava in Vojvodina (Serbia): right-biased directional asymmetry in *TA* and *J* was particularly high in males from these cemeteries (see Table 3 and Figure 3).

Vedrovice was located at the periphery of the earliest expansion of farming into Central Europe [114]–[116], with radiocarbon dates as early as ∼5480 cal BC [117], while the earliest date at Nitra is ∼5370 cal BC [118]. At this time in the Central European Early Neolithic, land was still relatively covered in primary forest or brush, requiring clearance prior to cultivation [48], [119]. This was likely accomplished through burning of the vegetation, but ground and polished stone adze-axe tools may also have been used for the clearance of trees, and hoes and digging sticks for the clearance of brush and digging of furrows. Axes and adzes would also have been required to procure and work materials in the construction of the timber longhouses that are characteristic of the early LBK cultural assemblage [48], [120]. The high mean %DA in humeral CSG properties among some males at these earliest LBK sites in Central Europe may be related to particularly strenuous use of adze-axe tools. The forces on the upper limb generated by their use are high, similar to those generated in sports requiring overhead throwing [121], and would certainly produce high unilateral loading in the dominant upper limb. Interestingly, though adult males at Stuttgart-Mühlhausen did not exhibit such pronounced right-lateralization in humeral CSG properties, there are indications that the LBK population at this site may have loaded their upper limbs to a greater extent than modern humans. Adults from Stuttgart-Mühlhausen had higher estimated mean forearm muscle cross-sectional areas (standardized by radius length) than a sample of 228 living Germans [122], which the authors interpreted as indicative of higher mechanical loading and significantly higher muscular activity of the forearm in these early LBK adults relative to modern humans.

Male mean %DA values in *TA* and *J* were also particularly high at the Vinča site of Gomolava (Middle Neolithic layers) relative to the Linear Pottery sites of the Early Neolithic (see Table 3 and Figure 3). There is evidence of copper metallurgy in the Vinča layers at Gomolava, one of very few known late Vinča cemeteries in Central Europe, including copper beads, bracelets, and chisels [123]–[125]. Mining and smelting of local copper ores in the Balkans dates back to the fifth millennium BC [126]–[127] and would have involved significant loading of the upper limbs. Ore had to be hammered out of the rock by hand using stone hammers and antler picks, then crushed up using smaller hand-held hammers, pestles, and pebble tools, before being roasted, hammered, and annealed at very high temperatures in shaft furnaces [78]. The extent to which Gomolava Vinča males were participating in copper metallurgy, if at all, is unknown, but the types of activities that produced high unilateral loading were present at this time, such as those associated with the mining, smelting, and production of copper objects, and these tasks were likely male-dominated.

With the Early Bronze Age, significantly higher %DA in *TA* and *J* in males relative to females suggests a clear difference in the degree of symmetrical versus asymmetrical upper limb loading between the sexes at this time. This pattern was driven primarily by a reduction in the proportion of females at the high end of %DA values relative to the Neolithic (see Fig. 2b); the ways that women's behaviors loaded their upper limbs changed in the Bronze Age to a greater extent than did those of men. The proportion of Bronze Age females performing highly right-biased loading declined relative to the Neolithic, indicative of greater task specialization among females and/or a change in the types of activities performed. Females from the Middle Bronze Age Füzesabony cemetery of Polgár Kenderföld (Hungary) [87] displayed, on average, almost completely symmetrical humeral *TA* and *J* (see Table 3 and Fig. 3).

Not only was loading symmetrical or even left-biased among Bronze Age females but right-lateralized among Bronze Age males, this symmetrical female loading was associated with substantially less variability in CSG properties, driving a reversal in the sexual dimorphism of humeral variation relative to the Neolithic period (Fig. 6). These upper limb patterns suggest that manual activities became more homogeneous among females in the Bronze Age relative to the Neolithic, with fewer females participating in strenuous right-biased unilateral tasks. Instead, the majority of Bronze Age females appear to have been participating in bilateral manipulative tasks, perhaps the sorting of crushed ores prior to smelting [77], agricultural fieldwork, the grinding of grains or ores, and pottery and textile fabrication, which loaded the upper limbs in similar and symmetrical or left-biased ways. In contrast, high %DA among Bronze Age males suggests that they continued to be regularly performing strenuous activities in which the right upper limb predominated; these could have included percussive hammering and pounding during mining, smelting, and smithing, as well as agricultural production and the use of unilateral weapons.

The most pronounced sex differences in humeral %DA were found in the Early Bronze Age Únětice settlement and burial site of Brno-Tuřany (Moravia, Czech Republic), where male %DA in humeral *J* was significantly higher than the slightly later Bronze Age sites examined and more than nine times greater than mean female values from the same site (see Table 3 and Fig. 3). Archaeological and anthropological details of the site of Brno-Tuřany were published in 2008 [80]. The site dates to the Moravian Early Bronze Age (approximately 2300-1700 BC) [54], a time of major metallurgical expansion and social change [79]. Archaeological finds from the site include bronze hair ornaments and pottery fragments typical of the Únětice culture [80]. Únětice graves across Central Europe often contain flint arrowheads and many metal weapons, such as daggers, flanged axes, and halberds, indicative of metalworking and weapons use, likely by males, as well as evidence of spinning, weaving, and pottery, tasks that are typically female-dominated [88]. It is likely that some of these activities were being performed at Brno-Tuřany. A similar pattern of %DA by sex (high in males and low in females) was identified by Sládek and colleagues [47] in an Early Bronze Age sample that included Únětice skeletal remains from Central Europe: mean female %DA in humeral *J* was very low (1.06%; below female means in the current study) compared to that of males (12.78%; similar to male means in the current study). The authors attributed this sex-specific pattern of asymmetry in the Early Bronze Age to the performance of domestic labor among females and their decreased participation in intensive agriculture.

Sex differences in percent right bias for humeral CSG properties were most pronounced in the Bronze Age (Fig. 4a,b), where 96% of males exhibited right dominance in *TA/J*, compared to just 65% and 73% of females in the same properties. Humeral CSG properties were consistently right biased in both sexes in all Central European agriculturalists studied, but male values in all time periods fall at the top of the range of modern human values (the high end of reported right-biasing in modern human humeri is ∼90%+) [13] and females at the bottom (the low end of reported right-biasing in modern humans is ∼75%) [28]. For instance, all males combined (N = 85) demonstrated an overall right bias of 91.8% in humeral *J*, while in all females combined (N = 71) the overall right bias was just 73.2%.

Maximum lengths exhibited much lower variability and asymmetry than CSG properties, though they were consistently more right-biased in females across both the Neolithic and the Bronze Age. This was despite significantly higher %DAs in CSG properties in Bronze Age males relative to females, so asymmetry in bone length in the Central European groups studied does not immediately appear to reflect the influence of manipulative behavior to any large degree. Similarly, Auerbach and Ruff [30] found no significant correlation between asymmetries in length and diaphyseal breadth in a wide variety of Holocene humans.

The current study and those of both Auerbach and Ruff [30] and Sládek and colleagues [47] all noted a similar reversed pattern of sexual dimorphism in directional asymmetry between humeral CSG properties and maximum length: right-biased CSG properties among males were associated with more symmetrical lengths, and vice versa among females. Strenuous and repetitive movements of the dominant upper limb practiced by athletes in overhead throwing/hitting sports, such as volleyball, baseball, and tennis, have been associated with changes in humeral torsion [128]–[132] and/or lateral deviations of the distal humerus (valgus deformity) [133]. Thus, it is possible that higher %DA in CSG properties combined with lower %DA in lengths may be reflecting some influence of mechanical loading on deviation or rotation in the humeral shaft that would indirectly affect length. Additional factors such as genetic predisposition, sex differences in growth, or fluctuating asymmetry could also be influencing bone length asymmetry in Central European agriculturalists.

### Bronze Age to Iron Age transition

The Neolithic through Bronze Age transition in Central Europe was characterized by divergence in the lateralization of upper limb loading, pronounced sexual dimorphism, and change in the range and type of manual activities performed by males and females. In contrast, the Bronze Age to Iron Age transition in Central Europe was typified by consistent and significant reductions in the sexual dimorphism that was characteristic of the Bronze Age. The current study suggests that the consistency of humeral loading from the Late Eneolithic to Early Bronze Age noted by Sládek and colleagues [47] extended into the Iron Age in this region, but only among males. Lateralization in male humeral CSG properties was not significantly affected by the incorporation of iron mining and smelting, iron tool manufacture and use, or social change. In contrast, Iron Age females exhibited significant increases in %DA in *TA* and *J* relative to the more symmetrical values in Bronze Age females (Fig. 2a,b), again suggesting that behavioral change through time affected the upper limb loading of women more than that of men.

Though Iron Age sample sizes were small, results by cemetery documented a consistent trend towards increasing mean %DA in humeral *TA* and *J* among females from the Early Bronze Age through Middle Iron Age (∼2300 BC to 200 AD; see Table 3 and Fig. 3). Based on a range of grave goods associated with the Iron Age in Central Europe [89] and published finds from Gomolava and Tápiószele [134]–[139], it is probable that Iron Age females were performing a range of manipulative activities associated with agricultural activities and livestock tending, the production of food, dairy products, textiles, clothing, personal ornamentation, and pottery, and various other domestic activities. Iron Age female humeral CSG properties were not significantly more variable than those of Bronze Age females in either humerus (see Table 5), although sample sizes were small and limit the ability to draw conclusions from this result. In any case, Iron Age females appear to have loaded their upper limbs significantly more unilaterally than Early/Middle Bronze Age females, who appear to have been primarily loading their upper limbs symmetrically.

Lateralized behaviors were particularly interesting in one small region of Moravia near Brno, Czech Republic: not only did Early Bronze Age males at Brno-Tuřany (Únětice) exhibit the highest %DA in humeral *J* of all groups sampled (19.16%), but over the 1300+ years between the use of Brno-Tuřany and Brno-Maloměřice (Celtic) [140]–[143], average female %DA in both *TA* and *J* increased by approximately eight-fold (see Fig. 3 for *J*), though sample sizes are very small. Decorated bronze artifacts recovered from Brno-Maloměřice have been well-documented [140], [144]–[145], and the cemetery is the largest Celtic burial ground in Moravia [146]. A larger sample size is required in order to determine whether or not this high %DA in the three females examined from Brno-Maloměřice was actually the norm at this site, but results suggest the possibility of population discontinuity and/or particularly complex changes in division of labor and social structure in Moravian metallurgical societies.

Significant Neolithic and Bronze Age sexual dimorphism in humeral maximum length asymmetry (more right-biased in females) also declined in the Iron Age, through a significant increase in male length %DAs relative to both preceding periods. It is not likely that this change in length asymmetry reflects major changes in the degree of upper limb loading in Iron Age males relative to previous males, as change in the lateralization of CSG properties would also be expected if this were the case. However, high task specialization among Iron Age males may have reduced the proportion of the population for whom activities would have involved the repetitive and strenuous overhead rotational motion (e.g., carpentry, mining, blacksmithing) that may have been influencing length asymmetry in earlier males.

## Conclusion

Comparisons of humeral cross-sectional geometry among 174 adults spanning ∼5400 years from the onset of agriculture in Central Europe found that the long-term social change, increasing task specialization, and the expansion of copper and bronze metallurgy were associated with little change in upper limb asymmetry and variation among males but considerable change among females. These temporal patterns are suggestive of major changes in the sexual division of labor and the range of manual activities performed by women through the first ∼5400 years of farming in Central Europe. A major divergence in the lateralization of upper limb loading between the sexes occurred in the Early Bronze Age: female activities that loaded the upper limbs became more symmetrical and homogeneous relative to the Neolithic. This may be related to changing agricultural activities among females with the introduction of the ard and plow and/or the increased importance of bilaterally symmetrical tasks such as the grinding of grain, weaving and textile production, and the preparation of ground ores for smelting. Female upper limb loading became significantly more right-biased in the Iron Age, likely associated with the performance of activities involving significant amounts of unilateral loading. In contrast, males through the entire Central European sample exhibited consistent right-biased upper limb lateralization, indicative of the predominance of unilateral upper limb loading. In the Neolithic, habitual behaviors producing this type of loading likely included the use of adzes and axes for woodworking and land clearance, while in the Bronze and Iron Ages, additional male-dominated unilateral activities would have included the mining of metal ores and the smelting and production of metal objects, as well as the use of weapons. In contrast to diaphyseal cross-sectional geometry, asymmetry and variability in bone lengths was minimal and was not clearly associated with mechanical loading.

## Supporting Information

Table S1 **Sample details and humeral percent directional asymmetries in** ***TA*** **,** ***J*** **, and maximum length for all individuals.**
(XLSX)

Table S2 **Sample details and size-standardized** ***TA*** **,** ***J*** **, and maximum lengths of the left and right humeri for all individuals.**
(XLSX)
